# Esterase D and gamma 1 actin level might predict results of induction therapy in patients with acute myeloid leukemia without and with maturation

**DOI:** 10.1007/s12032-013-0725-2

**Published:** 2013-10-02

**Authors:** Maciej Kaźmierczak, Magdalena Luczak, Krzysztof Lewandowski, Luiza Handschuh, Anna Czyż, Małgorzata Jarmuż, Michał Gniot, Michał Michalak, Marek Figlerowicz, Mieczysław Komarnicki

**Affiliations:** 1 Department of Hematology, Poznan University of Medical Sciences, Szamarzewskiego 84, 60-569 Poznań, Poland; 2 Institute of Bioorganic Chemistry, Polish Academy of Sciences, Noskowskiego 12/14, 61-704 Poznań, Poland; 3 Department of Computer Science and Statistics, Poznan University of Medical Sciences, Dąbrowskiego 79, 60-529 Poznań, Poland; 4 Institute of Computing Science, Poznań University of Technology, Piotrowo 3A, 60-965 Poznań, Poland

**Keywords:** Acute myeloid leukemia, Proteome profiling, Mass spectrometry, Esterase D, Gamma 1 actin, Outcome prognosis

## Abstract

Development of modern proteomic methods in recent years has opened also new perspectives in the identification of new biomarkers which ensure more effective diagnosis, treatment monitoring and prediction of therapeutic outcome. We evaluated usefulness of comparative proteomics (MALDI-TOF) in two subtypes of acute myeloid leukemia (AML), M1 and M2, according to FAB classification. The bone marrow or blood cell proteomes were examined in 33 newly diagnosed patients before “3 + 7” induction therapy, after treatment and when the disease relapsed. We found that bone marrow and peripheral mononuclear cells from healthy volunteers revealed a number of quantitative and qualitative differences between the two proteomes, reflecting differences in the maturational status of normal cells. Such differences were not detected in our AML M1/M2 patients. Additionally, we found 9 proteins, which are potential biomarkers differentiating between the AML patients and healthy volunteers. Using comparative proteomics, we found that annexin I, glutathione transferase omega, esterase D and gamma 1 actin had prognostic significance. Applying statistical methods, we detected two proteins which might allow to predict results of induction therapy in AML M1/M2. One of them was esterase D, the higher concentration of which was associated with higher complete remission rate, and the other was gamma 1 actin, the higher concentration of which was related to resistance. In the article, we also discussed the role of these two proteins in the biology of AML, and we suggested potential usefulness of modification in induction therapy reflecting the presence of proteins.

## Introduction

Acute myeloid leukemia (AML) is a heterogenous clonal disorder of either multipotent hematopoietic stem cells (HSCs) or more mature committed myeloid precursors downstream of HSCs [1]. Clonal cytogenetic alterations are present in AML in more than 50 % of adults and are considered the strongest predictor of response to the therapy and overall survival (OS). Identification of specific gene abnormalities (e.g., *FLT3*, *NPM1*, *CEBPA*) improved prognostic allocation especially in the group of patients with the, so-called, normal cytogenetics. During the last decade, DNA microarrays were extensively used to identify genes involved in pathogenesis of human myeloid malignancies [2]. The gene expression profiles were determined for all AML subtypes [3–5]. Unfortunately, the transcriptomic analyses did not lead to a real breakthrough in our understanding of the molecular mechanisms underlying AML development and the reason of the therapy failure.

As there is still a large group of AML patients for which the prediction of disease outcome and response to the treatment is very difficult or even impossible, therefore, there is an immense need for new methods and biomarkers which may enable better differentiation between various AML subtypes and specification of their biological characteristics. One of them could be proteomics. So far, most of the comparative proteomic analyses involving acute leukemia patients were focused on identification of proteins which can be used to distinguish different morphological subtypes and on the correlation between the profile of protein accumulation and AML karyotype [6, 7]. Protein microarray-based studies represent another interesting attempt to identify FAB subtype-specific AML biomarkers. Kornblau and coworkers selected proteins, which can distinguish subtypes of AML and predict the results of treatment. They defined 7 protein signature groups, with prognostic information distinct from that provided by cytogenetics [8].

Recently, we have carried out a detailed proteomic analysis of bone marrow and peripheral blood mononuclear cells obtained from AML M1 and M2 (AML without and with maturation) patients. As a result, we have identified proteins, the accumulation profiles of which were different in the studied AML subtypes. Accordingly, we have postulated that these proteins can be classified as potential biomarkers [9]. In order to verify this hypothesis, herein we present a consecutive analysis of the selected proteomic and clinical data obtained for the same group of patients. In addition, we discuss the possibilities of using comparative proteomics in AML diagnosis and in monitoring of the treatment.

## Materials and methods

### Study population

The studies involved consecutive 38 AML M1 or M2 patients (diagnosed according to the FAB classification) admitted to the Department of Hematology, Poznan University of Medical Sciences between February 23, 2007, and November 19, 2009. Because of incomplete clinical data in 5 cases, we qualified 33 patients to final analysis. The last follow-up was made in April 2012. Among the 33 newly diagnosed patients, 30 were treated with the standard “3 + 7” induction therapy (cytarabine/Ara-C/and doxorubicine). Three patients died before the treatment were applied. The results of chemotherapy, defined by European LeukemiaNet, were established only in 25 cases because 5 patients died during and up to +7 days after the induction therapy [10]. In this group, 18 patients (72 %) achieved complete remission (CR) and 7 were resistant (RES). As a consolidation therapy, the high-dose Ara-C alone (HDAra-C) or combined with anthracycline (HAM) and hematopoietic stem cell transplantation were used (HSCT; 5 allogeneic/alloHSCT/and 1 autologous/autoHSCT/). From 7 resistant patients, only 1 achieved CR after administration of FLAG polychemotherapy (HDAraC, fludarabine and G-CSF). The samples of blood and bone marrow were collected at the following time points (if possible): when AML was diagnosed (T0), when CR was established between day +21 and day +28 after start of induction therapy (T1) and at the moment when the disease relapsed (T2). In all AML M1 patients, the isolated fractions of blood and bone marrow mononuclear cells contained more than 90 % of leukemic cells, and in most AML M2 patients, these fractions contained about 70 % of blasts. For a more detailed patient description, see Table 1. The control group consisted of 17 healthy volunteers (HV). In this case, investigational material consisted of 4 samples of bone marrow, collected during harvesting of cells for alloHSCT, and 13 samples of peripheral blood. The study conformed to the ethical guidelines of the World Medical Association Declaration of Helsinki. Each patient and healthy volunteer provided signed informed consent for treatment and participation in this study. Before commencement of the project, appropriate approval was obtained from the Bioethical Commission of Poznan University of Medical Sciences.

**Table 1:** Clinical characteristics of patients with acute myeloid leukemia (AML) M1 and M2 according to FAB classification treated with “3 + 7” induction therapy

Variables	No. of patients
Total number of patients	33
Diagnosis	
AML M1	11
AML M2	22
Sex	
Female	14
Male	19
Age (year, median, range)	52 (19–65)
To 59 years	26
60 years and more	7
General status by ECOG	
0–1	26
Over 1	7
Accompanied diseases	
Cardiac	15
Other	4
Malignant diseases in the past	2
Cytogenetic risk by SWOG	
Favorable	2
Intermediate (all patients with normal karyotype)	13
Unfavorable	4
Unknown	14
Mutational analysis	
AML-ETO	5
FLT3	4
Number of leukocytes (G/L)	
Below 4,0	10
4.0–30.0	8
Over 30.0–100.0	10
Over 100.0	5
LDH over range	16
Myelodysplasia	3
Death before induction therapy	3
Induction therapy “3 + 7”	30
Results after first induction therapy	
CR	18 (72 %)
Resistance	7 (28 %)
Death during and up to +7 day after induction therapy	5
Death after induction therapy (from +8 day)	4 (with resistance)
Results after second induction therapy	
CR	1
Resistance	2
Consolidation therapy	
1 course	5
2 courses	8
3 courses	5
Median number of courses	2
HSCT	
Allo	5
Auto	1
Relapse	10 (53 %)
Survival time in first CR (months, median, range)	10 (4–34)
To 12 months	13
Over 12 months	6
Survival time in first and second CR (months, median, range)	12 (7–52)
To 12 months	9
Over 12 months	10
Overall survival in group of 33 patients (months, median, range)	8 (0–52)
Overall survival in group of 19 patients with CR (months, median, range)	12 (7–52)
Still alive	7 (37 %)

### Samples collection and processing

Peripheral blood and/or bone marrow samples were collected into a closed monovette system containing EDTA anticoagulant. Mononuclear cells were isolated by Gradisol density-gradient centrifugation, and thereafter, they were washed with PBS. Then, total proteins were isolated from mononuclear cells using *mir*Vana™ Ambion Isolation Kit (according to the protocol provided by the manufacturer). In addition, for all AML patients, the accumulation of *AML1*-*ETO* [also known as *RUNX1/RUNX1T1;* indicative of t(8;21)(q22;q22)] and *FLT3*-*ITD* in mononuclear cells was determined by RT-PCR. Cytogenetic analysis was performed on metaphases from samples of bone marrow obtained prior to induction therapy (T0) by using standard banding techniques. Karyotypes (GTG) were determined according to the International System for Cytogenetic Nomenclature [11].

**Fig. 1:**
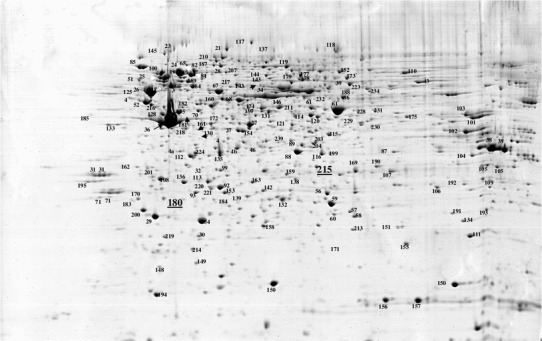
A representative example of the 2D PAGE analysis of bone marrow samples collected from patients with acute myeloid leukemia M1 and M2 according to FAB classification. The proteins identified by mass spectrometry are indexed by numbers (esterase D–spot 215, gamma 1 actin–spot 180)

All detailed methods concerning 2D electrophoresis, gel image analysis, protein identification by mass spectrometry (MALDI-TOF or ESI–MS/MS) were presented in the former paper of the same authors (Fig. 1) [9]. The statistical methods used for analysis of proteomic results indicated four proteins (annexin I, glutathione transferase omega, esterase D/formylglutathione hydrolase/and gamma 1 actin) with the best prognostic significance. In this paper, we attempted to correlate the presence of these proteins with the entire set of clinical data.

### Statistical analysis

The relationship between clinical parameters and results of induction chemotherapy (CR or RES) was analyzed by Fisher’s exact test. Univariate analysis of the major outcome variables and the protein concentrations (annexin I, glutathione transferase omega, esterase D and gamma 1 actin) was performed using logistic regression. Factors for which the *p*-value was < 0.05 in the univariate analysis were included into a multivariate logistic regression model. Adjusted odds ratios (OR) and 95 % confidence intervals were calculated.

Receiver operating characteristics (ROC) curves were calculated to determine the potential of analyzed factors to discriminate the result of induction therapy. An optimal cut-off point was calculated according to the highest accuracy (minimum false negative and false positive results). The area under the ROC curve (AUC) was used to check the prognostic value of a particular variable. The tests were considered statistically significant at *p* < 0.05. Statistical analyses were performed using Statistica 10 (StatSoft Inc., Poland) software and MedCalc version 10.3.2 (MedCalc Software, Mariakerke, Belgium).

## Results

### Proteomic analysis of bone marrow (BMMC) and peripheral blood (PBMC) mononuclear cells in AML patients and HV [9]

The comparison between AML-BMMC and AML-PBMC proteomes at T0, T1 and T2 did not show any statistically significant differences between them, and therefore, data for these samples were calculated together. On the other hand, a comparative analysis of BMMC and PBMC from HV revealed numerous quantitative and qualitative differences between the two proteomes. The set of proteins differentiating them was finally limited to 28, which matched the established criteria (we excluded extracellular proteins that were found to be contaminants or proteins for which the relative accumulation levels were below 0.004).

Comparative analysis of AML M1/M2 and control BM/PB cells revealed 25 differentially accumulating proteins. They included 8 proteins (catalase, tumor rejection antigen/Gp96/, tubulin β, vinculin, peroxiredoxin-2, purine nucleoside phosphorylase, annexin 1 and protein disulfide-isomerase), which simultaneously differentiated healthy bone marrow/blood mononuclear cells from AML M1/M2 mononuclear cells and healthy blood cells from healthy bone marrow. Additionally, we found 9 proteins (histone-binding protein RBBP4, α-actinin 1, 14-3-3 protein, transketolase, pyruvate kinase, DJ-1 protein, F-actin capping protein alpha-1, protein PP4-X and moesin), which represented potential biomarkers differentiating between the AML M1/M2 patients and healthy volunteers.

### Differences between acute myeloid leukemia without (AML M1) and with maturation (AML M2) at the proteome level [9]

We used a function of the Image Master 2D Platinum 6.0 program to distinguish between two subtypes of AML according to FAB classification–M1 and M2 at proteome level. We found that AML M1/M2-T0 group can be divided into 2 subgroups. The important fact was that samples from the same patients (BMMCs and PBMCs) were always in the same subgroup. Moreover, all samples from patients with the diagnosed *t*(8,21) (q22; q22) were in the same subgroup, defined as AML M2. There were no differences in protein accumulation between patients with or without this translocation in the group of AML M2 patients. Next, we performed a comparative proteomic analysis within the AML M1-T0 and AML M2-T0 subgroups. As a result, we identified 5 proteins differentially accumulating in two subgroups. Three from those proteins—annexin III, L-plastin isoform and 6-phosphogluconate dehydrogenase—were detected only in the AML M2-T0 group. Catalase and peroxiredoxin 6, on the other hand, were manifested in both analyzed groups; however, they showed a higher level of concentration in the AML M2-T0 group.

We also found that such proteomic-based classification was in 88 % consistent with the clinical diagnosis, based on cytomorphology, immunophenotyping and peroxidase reaction.

### Proteomic analysis of blood and bone marrow cell samples after induction chemotherapy and at the time of relapse [9]

We compared the proteomes of individual patients before treatment (T0) and after treatment, when CR was obtained (T1), and we did not succeed in identifying any proteins, which would differentiate both groups. The results obtained showed that in case of samples collected at T1, we dealt with a highly diversified pool of cells. Their proteome exhibited very high individual variability.

In addition, we attempted to determine whether at the time of relapse (T2), the proteome of mononuclear leukemic cells is similar to or different from the proteome of the cells collected at T0. The analyses did not reveal any statistically significant differences. Therefore, we suggested that the proteome of cells before treatment and at relapse was similar, at least at the level of highly abundant proteins.

### Correlation of clinical/proteomic characteristics and results of treatment (complete remission or resistance)

No dependence between results of treatment and traditional clinical characteristics—age, sex, subtype of AML, performance status, history of prior malignancy and chemotherapy, dysplasia, infection status, cardiac status, white blood cell count, hemoglobin, platelet count, percentage of blood and bone marrow blasts, percentage cells expressing CD 7, 11b, 14, 34, 56, HLA-DR, creatinine and bilirubin concentration in blood, cytogenetics, molecular findings was observed (data not presented).

On the basis of proteomic analysis of blood and bone marrow samples from patients with AML before induction therapy (T0) who achieved complete remission or were resistant, we found four proteins, the presence of which significantly correlated with results of treatment. The prognostic significance was manifested by annexin I, glutathione transferase omega, esterase D/formylglutathione hydrolase/and gamma 1 actin (Table 2). Two proteins (glutathione transferase omega and esterase D) were detected only in samples from AML M1/M2 patients with CR. Annexin I was present in both subgroups, however, its concentrations were significantly higher (*p* < 0.001) in the subgroup with CR (a threefold difference). Gamma 1 actin accumulated to a significantly higher level (a 2.85-fold difference) in mononuclear cells in AML M1/M2-T0 subgroup with resistance to induction therapy and also in patients with short-term remission (below 12 months). Additionally, results obtained for annexin I and gamma 1 actin were verified using the Western blot method.

**Table 2:** ROC analysis of protein concentrations which significantly discriminate results of “3 + 7” induction therapy in patients with acute myeloid leukemia with and without maturation (complete remission or resistance)

Protein	AUC	*p* value	Cut-off point	Sensitivity %	Specificity %
Annexin I	0.77	*p* = 0.0024	>0.4676	77.8	85.7
Esterase D	0.84	*p* < 0.0001	>0	88.9	85.7
Glutathione transferase omega	0.94	*p* < 0.0001	>0	88.9	100
Gamma 1 actin	0.79	*p* = 0.0084	≤0.2087	72	100

Glutathione transferase omega manifested the best prognostic value; but on the basis of logistic regression analysis, we identified two other proteins, esterase D and gamma 1 actin, which provided the best index pointing to a complete remission (*p* = 0.0032). An increase in esterase D concentration by 1 unit augmented about 10^15^-fold the probability of CR. On the other hand, an increase in gamma1 actin concentration by 1 unit multiplied the probability of RES by about 10^3^. This statistical model gave correct prediction in 92 % of cases. Cross-validation gave 94.4 % accuracy for CR results and 85.7 % accuracy for RES results.

## Discussion

The dynamic development of modern proteomic methods observed in recent years has opened new perspectives in the research on mechanisms underlying leukemic transformation. Special attention is paid to the identification of new biomarkers which ensure more effective diagnosis, treatment monitoring and prediction of therapeutic outcome [12]. We have decided to evaluate usefulness of comparative proteomics in AML, and we have focused on two subtypes M1 and M2 according to FAB classification. The bone marrow or blood cell proteomes were collected from patients before treatment (T0), when CR was obtained after induction therapy (T1) and when the disease relapsed (T2). Using 2D electrophoresis and mass spectrometry, we have found that BM and PB mononuclear cells from healthy volunteers revealed a number of quantitative and qualitative differences between the two proteomes, reflecting differences in the maturational status of normal cells [9]. On the other hand, however, we have detected no significant differences in the proteomes of bone marrow and peripheral blood mononuclear AML cells, which concurs with other authors’ opinions [8, 9]. Comparative analysis of AML M1/M2 and control PB/BM cells has revealed 25 differentially accumulating proteins. We have found 9 proteins, which might serve as potential biomarkers, differentiating between the AML M1/M2 patients and healthy volunteers [9]. It means that almost in all cases, it has been possible to diagnose and discriminate AML M1 and M2 only on the basis of peripheral blood analysis. It might be very useful, but classification systems like FAB evolved from it; the WHO classification still is based on cytomorphology, cytochemistry and also on immunophenotypic, cytogenetic, molecular features [13]. Some attempts are being made to introduce proteomics into clinical diagnostics in the broad sense [14, 15]. Researchers dealing with this problem pay particular attention to the necessity of selection, verification and determination of the so-called standard operational procedures (SOP). Therefore, we think that in this field, proteomics could not replace standard evaluations.

The available diagnostic methods do not always make it possible to distinguish between AML-M1 and M2 in an unambiguous manner. Therefore, we have decided to find out whether differences in the proteome of mononuclear cells justify the distinction of the two disease subtypes in the FAB classification. Hierarchical clustering of proteomic results has clearly divided AML samples into 2 groups (M1 and M2). Moreover, all samples from patients with diagnosed t(8,21)(q22;q22) translocation have fitted the same subgroup defined as AML M2. There exist no differences in protein accumulation between patients with or without this translocation in group of AML M2 patients [9]. Some authors found that sorcin, calcium-binding protein, was characteristic for patients with such translocations [7]. We have also showed that such “proteomic classification” was in 88 % consistent with the clinical diagnosis, based on cytomorphology, immunophenotyping and peroxidase reaction. From the practical point of view, there is no difference in therapy and prognosis of AML M1 or M2. Only the presence of isolated t(8,21)(q22; q22) influences the postremission therapy. We have compared the proteomes of individual patients in T0 and T1, and in each case, we have found several differentiating proteins, but the differences have proven to be insignificant. The obtained results have shown that in case of samples collected after treatment, we dealt with highly diversified pools of cells. Their proteome has exhibited very high individual variability. In addition, we have tried to determine differences in proteome before treatment and at the time of relapse. Due to the fact that the analyses have revealed no significant differences between them, we suggest that the proteome of cells has remained similar, at least at the level of highly abundant proteins [9].

In adult patients with acute myeloid leukemia (without acute promyelocytic leukemia), the use of intense induction chemotherapy “3 + 7” is the worldwide standard of care. Its administration allowed to obtain CR in approximately 70–80 % of patients under 60 years of age. However, most of them relapse, and therefore, overall survival in this group is only 40–45 % at 5 years. The results of therapy are much worse in the group of older patients (over 60 years old) [16]. Due to generally unsatisfactory results, in the recent years, there have been several practice-changing developments in the diagnosis and treatment of acute myeloid leukemia. Clonal chromosome alterations are universally considered to represent the strongest predictor of duration of response and overall survival [17]. Progress in genomic technologies has identified AML, especially that with a normal karyotype, as a genetically highly heterogeneous disease, and an increasing number of AML patients can now be categorized into distinct clinico-pathologic subgroups on the basis of their underlying molecular genetic defects [18]. We must remember that fitting the increasing number of new gene abnormalities into a prognostic algorithm is a very difficult task. A reasonable way to improve outcome prediction in AML might be attained by combining pretreatment and posttreatment parameters into a common prognostic algorithm [19].

In this paper, authors have focused on the pretreatment prognostic factors. On the one hand, we have attempted to evaluate influence of clinical, cytogenetic, molecular data on results of chemotherapy. On the other hand, we have correlated all the mentioned above data with results of comparative proteomics to establish its usefulness to foresee the results of induction therapy (complete remission or resistance).

The studied group of patients with AML M1 and M2 has consisted mostly of younger people (below 60 years old) in good performance status. The statistical analysis of traditional clinical characteristics—age, sex, subtype of AML, performance status, history of prior malignancy and chemotherapy, dysplasia, infection status, cardiac status, white blood cell count, hemoglobin level, platelet count, percent blood and marrow blasts, percent cells expressing CD 7, 11b, 14, 34, 56, HLADR, creatinine and bilirubin concentration, cytogenetics, molecular findings and results of induction chemotherapy (“3 + 7”) has demonstrated no significant correlations. We are aware that our study group was too small to draw final conclusions in this subject. However, we have performed also very initial survival analyses, and we have found that favorable cytogenetics (*p* = 0.048) and consolidation therapy based on alloHSCT (*p* = 0.019) prolonged overall survival (OS). Meanwhile, occurrence of disease relapse (*p* = 0.043) and cardiologic side effects (0.024) has shortened OS.

Using comparative proteomics, we have found that annexin I, glutathione transferase omega, esterase D/formylglutathione hydrolase/and gamma 1 actin have manifested a prognostic significance. Applying statistical methods, we have selected two proteins which might provide new biomarkers in prognosis of clinical behavior in AML, M1 and M2, according FAB classification. These are esterase D and gamma 1 actin. Higher concentration of the first protein seems to correlate with high probability of obtaining complete remission. On the contrary, a high concentration of the second protein seems to be related to a high probability of resistance to chemotherapy. The mentioned above biomarkers are distinct than those presented by other authors. Kornblau et al. identified a combination of mutant p53 and high levels of MCL1 and NRP1 as an adverse prognostic combination, associated with the lower remission rates, highest relapse and worse survival. One of possible explanations of the difference might involve the type of employed diagnostic methods. We have used comparative proteomics (MALDI-TOF) while Kornblau et al. [8] assayed AML cells for 51 total and phosphoproteins using reverse-phase protein arrays (RPPA).

The first proteomic biomarker proposed by us is esterase D, also known as S-formylglutathione hydrolase. The enzyme is involved in detoxification of formaldehyde [20]. However, the precise biological function and physiological role of ESD still remain unclear. Genetic polymorphism of esterase D (ESD) and its reduced enzymatic activity was found to be associated with the susceptibility to several pathological conditions like toxic liver cirrhosis, retinoblastoma, Wilson’s disease, obesity and autism. Recently, Wiedl et al. [21] found that a decreased activity of esterase D predicts development of a more aggressive course of the human lung adenocarcinoma with distant metastases. The same correlation, like in lung adenocarcinoma, we observed in our group of patients.

In our opinion, the second protein which potentially carries a prognostic value in AML M1 and M2 patients is gamma 1 actin. Actins are an essential component of the cytoskeleton, with critical roles in a wide range of cellular processes, including cell migration, cell division and the regulation of gene expression [22]. An over-activated Rho-associated kinase (ROCK) signaling pathway was suggested in gamma actin-knockdown cells. It means that gamma actin is a potential upstream regulator of ROCK-mediated cell migration. ROCK is an effector of the small GTPase Rho (mainly known for its involvement in cell adhesion, migration, proliferation and cell transformation). Activation of Rho or ROCK induces a sustained, but not transient, c-Jun NH2-terminal kinase (JNK) activation, which reduces the ability of cells to migrate and is associated with apoptosis [23, 24]. The results of our research have shown that high concentration of the gamma 1 actin may be a negative prognostic factor, predicting resistance. It is likely to occur by inhibition of Rho-ROCK-JNK axis. Such resistance to apoptosis was also suggested by Kornblau et al. [8] as a main reason of chemoresistance within FAB M0 to M2 leukemias. Our results may suggest that adding drugs which influence Rho-ROCK-JNK axis to the induction therapy could improve outcomes, especially in the group of patients with high level of gamma 1 actin at the time of diagnosis of AML. We propose arsenic trioxide (ATO), which induces apoptosis in this way [25]. Till now, ATO was used alone in a group of non-APL AML patients who were resistant to first-line therapy, who had secondary AML or whose age exceeded 65 years old. The study results were not satisfactory [26]. Gail et al. studied AML; non-APL patients over 60 years old were treated with ATO plus low-dose Ara-C. Such a combined therapy was more effective than that with Ara-C alone [27]. Other authors did not observe such a benefit [28]. Wentzler with coworkers presented improved results of induction therapy with ATO, HDAra-C and idarubicin in patients aged below 60 years with de novo AML compared with patients treated without ATO in a non-randomized comparison [29]. Although this improved outcome may be explained by earlier detection or improvements in supportive care over the periods when these 2 sequential studies were conducted, they proposed a different explanation. First, that ATO targets quiescent leukemia-initiating cells, and second, that combining ATO with Ara-C significantly increased its efficacy to induce apoptosis and eradicate the leukemia-initiating cells [30].

In conclusion, there are no doubts that esterase D and gamma 1 actin, suggested by the authors to represent prognostic factors involving results of induction therapy in acute myeloid leukemia M1 and M2, should provide targets of further more detailed investigations. We want to establish not only the utility of these proteins in prognosis of a complete remission or resistance in other subtypes of AML, but also in prognosis of the effects of combined induction therapy with or without arsenic trioxide in a group of patients with high gamma 1 actin level. We suggest that ATO represents a drug which could improve results of treatment in AML, but we think that also other drugs which influence Rho-ROCK-JNK axis deserve further investigation.
